# UvAtg8-Mediated Autophagy Regulates Fungal Growth, Stress Responses, Conidiation, and Pathogenesis in *Ustilaginoidea virens*

**DOI:** 10.1186/s12284-020-00418-z

**Published:** 2020-08-12

**Authors:** Shuai Meng, Meng Xiong, Jane Sadhna Jagernath, Congcong Wang, Jiehua Qiu, Huanbin Shi, Yanjun Kou

**Affiliations:** 1 State Key Laboratory of Rice Biology, China National Rice Research Institute, Hangzhou, 311400 China; 2 Key Laboratory of Three Gorges Regional Plant Genetics & Germplasm Enhancement (CTGU)/Biotechnology Research Center, China Three Gorges University, Yichang, 443000 China

**Keywords:** UvAtg8, Rice false smut, *Ustilaginoidea virens*, Autophagy, Secondary spore, Pathogenicity

## Abstract

**Background:**

*Ustilaginoidea virens* has become one of the most devastating rice pathogens in China, as well as other rice-growing areas. Autophagy is an important process in normal cell differentiation and development among various organisms. To date, there has been no optimized experimental system introduced for the study of autophagy in *U. virens*. In addition, the function of autophagy in pathogenesis remains unknown in *U. virens.* Therefore, the functional analyses of UvAtg8 may potentially shed some light on the regulatory mechanism and function of autophagy in *U. virens.*

**Results:**

In this study, we characterized the functions of UvAtg8, which is a homolog of *Saccharomyces cerevisiae* ScAtg8, in the rice false smut fungus *U. virens*. The results showed that *UvATG8* is essential for autophagy in *U. virens.* Also, the *GFP-UvATG8* strain, which could serve as an appropriate marker for monitoring autophagy in *U. virens,* was generated*.* Furthermore, this study found that the Δ*Uvatg8* mutant was defective in the vegetative growth, conidiation, adaption to oxidative, hyperosmotic, cell wall stresses, and production of toxic compounds. Pathogenicity assays indicated that deletion of *UvATG8* resulted in significant reduction in virulence of *U. virens*. Further microscopic examinations of the infection processes revealed that the severe virulence defects in the *∆Uvatg8* were mainly caused by the highly reduced conidiation and secondary spore formation.

**Conclusions:**

Our results indicated that the UvAtg8 is necessary for the fungal growth, stresses responses, conidiation, secondary spore formation, and pathogenicity of *U. virens*. Moreover, our research finding will potentially assist in further clarifying the molecular mechanism of *U. virens* infection, as well as provide a good marker for autophagy in *U. virens* and a good reference value for the further development of effective fungicides based on gene targeting.

## Introduction

Rice false smut disease is caused by the ascomycete fungal pathogen *Ustilaginoidea virens*. It is currently one of the most devastating rice fungal diseases in China, as well as many other countries (Fan et al. 2016; Jia et al. 2014; Ladhalakshmi et al. 2012; Nessa et al. 2015; Qiu et al. 2019). The typical and only visible symptom of rice false smut disease is the replacement of rice grains with false smut balls. The occurrence of rice false smut disease not only results in decrease of rice quality and serious loss of rice yield, but also threatens food safety due to its production of toxic mycotoxins (Lu et al. 2015; Lin et al. 2018; Meng et al. 2015; Wang et al. 2017; Sun et al. 2017). However, it has been found that the rice false smut disease is difficult to control. This is due to the fact that no resistant genes or completely immune rice varieties for rice false smut disease have yet been identified. Therefore, it is more and more important to deepen the understanding of the pathogenic mechanism of *U. virens* in order to provide clues for the development of more effective strategies to control the disease.

Autophagy is a tightly controlled catabolic cellular process which promotes homeostasis and adaptation by quality and quantity control of cytoplasmic components (Liu et al. 2016; Zong et al. 2016; Hofius et al. 2017). During the processes of autophagy, cytosolic materials, including damaged proteins and organelles, are captured by double membrane phagophore, which is followed by targeting to the vacuoles or lysosomes for degradation and recycling. After the degradation by resident hydrolases, the recycled components can be used for the synthesis of new molecules and/or providing energy source. Autophagy is involved in many important processes, including cell differentiation, development, and responses to nutrient starvation. The disruption of autophagy leads to impaired pathogenesis in some plant fungal pathogens (Sumita et al. 2017; Shi et al. 2019; Ren et al. 2018; Nadal and Gold 2010; Josefsen et al. 2012; Deng et al. 2009; Liu et al. 2016; Zhu et al. 2019).

Over the past two decades, the molecular mechanisms and machinery required for autophagosomal biogenesis and the subsequent degradation of cargo have been well characterized. In previous studies, more than 40 autophagy related (ATG) genes have been identified, which are necessary for this complex process (Liu et al. 2016; Zong et al. 2016; Hofius et al. 2017). Among those genes, *ATG8* (referred to as the LC3/GABARAP protein in humans), which encodes an ubiquitin-like protein, is a key component of the autophagy pathway (Ichimura et al. 2000). During autophagy, Atg8 is processed by the protease Atg4 and conjugated to the membrane lipid phosphatidylethanolamine (PE) through an ubiquitylation-like reaction, which is catalyzed by the E1-like enzyme Atg7, E2-like enzyme Atg3, and an E3-like complex Atg12-Atg5-Atg16 (Yoshimura et al. 2006; Ichimura et al. 2000; Hanada et al. 2007). The lipidated Atg8 mediates the tethering and hemifusion of membranes leading to autophagosome maturation (Nakatogawa et al. 2007).

In several plant pathogenic fungi, Atg8 is involved in various physiological processes, including cell differentiation, fungal development, secondary metabolism, and pathogenesis. In *Magnaporthe oryzae*, which is an important fungal pathogen of rice, Atg8-mediated autophagy is necessary for conidiation, formation of infection structure, and penetration (Veneault-Fourrey et al. 2006; Deng et al. 2009) (Veneault-Fourrey et al. 2006, Deng et al. 2009). For *Ustilago maydis*, which causes corn smut disease, Atg8 is required for proper morphogenesis and pathogenicity (Nadal and Gold 2010). In addition, deletion of *FgATG8* results in reduced hyphal growth, reproductive growth, and myctoxin biosynthesis in the destructive wheat pathogen *Fusarium graminearum* (Josefsen et al. 2012). In *Bipolaris maydis*, responsible for southern corn leaf blight, BmAtg8 plays a crucial role in the function of conidia, but not in the host infection via appressoria (Sumita et al. 2017). In *Botrytis cinerea*, the mutant lacking of *BcATG8* shows defects in fungal development, lipid metabolism, and pathogenesis (Ren et al. 2018). In the chest blight fungus *Cryphonectria parasitica*, *CpATG8* is not only required for pathogenesis but also is involved in hypovirus accumulation (Shi et al. 2019).

Despite the knowledge of autophagy and Atg8 in various fungi, its role in the rice fungal pathogen *U. virens* remains obscure. In this study, UvAtg8, a ScAtg8 homologue, was identified in *U. virens* and for the first time an experimental system was optimized to elucidate the function of autophagy in phytopathogenic *U. virens*. Our results indicated that UvAtg8 mediated autophagy played important roles in the growth, stresses responses, production of toxic compounds, conidiation, secondary spore formation, and pathogenicity of *U. virens*.

## Results

### Identification of UvAtg8

**Fig. 1:**
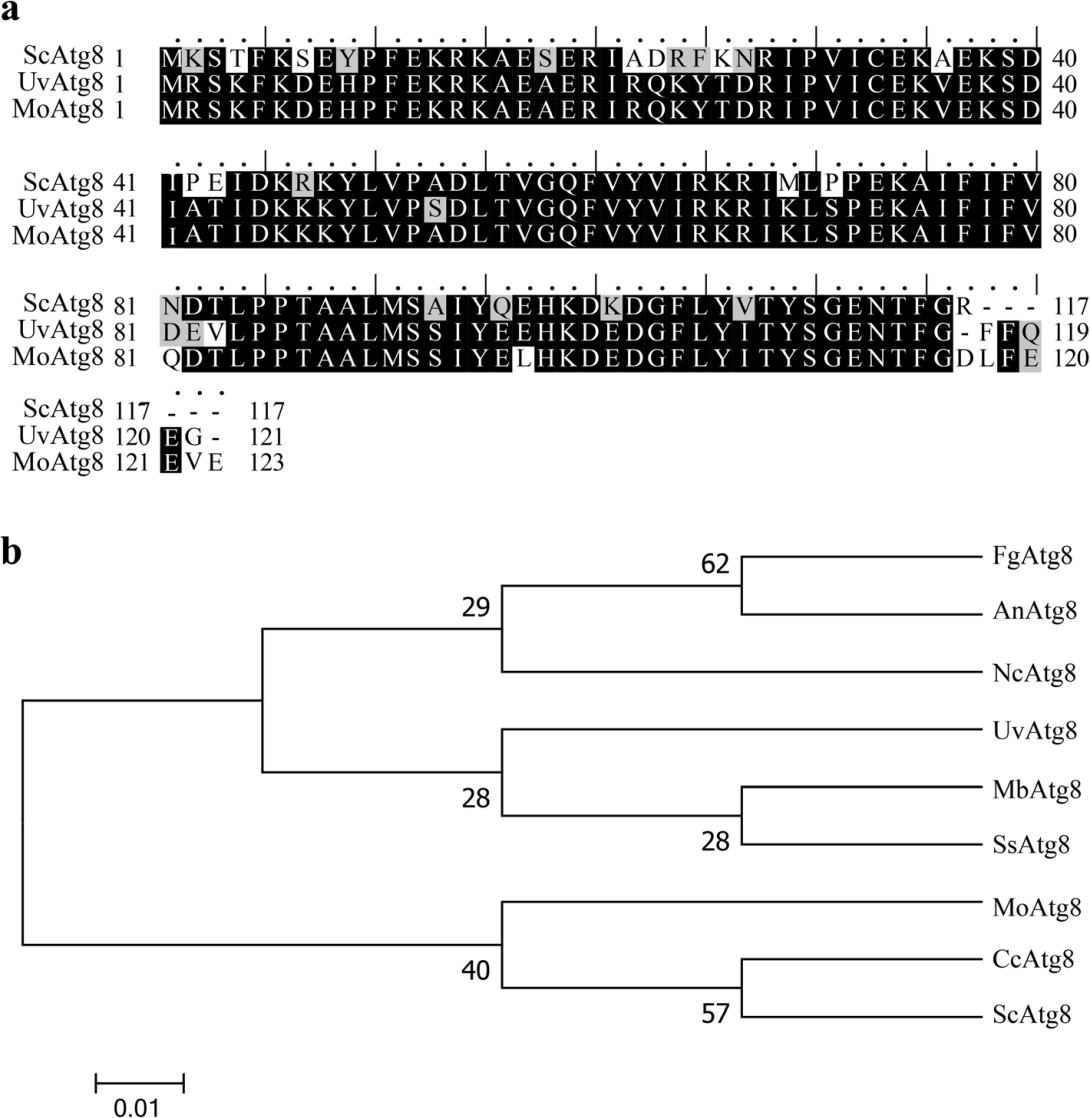
Evolution analysis of UvAtg8. a, The mutiple alignment of amino acid sequences of UvAtg8, ScAtg8, and MoAtg8. The identitical amino acid were shaded in solid black, and the amino acids with 50 % similarity were highlighted in gray. b, Phylogenetic tree of Atg8 orthologs from different fungi was constructed by MEGA 5.0 using the neighbour-joining method. The sequences included FgAtg8 (*Fusarium graminearum,* XP_001392077.1), AnAtg8 (*Aspergillus niger*, XP_001392077.1), NcAtg8 (*Neurospora crassa*, XP_956248.1), UvAtg8 (*Ustilaginoidea virens,* KDB12146.1), MbAtg8 (*Metarhizium brunneum*, XP_014545792.1), SsAtg8 (*Sclerotinia sclerotiorum*, XP_001597408.1), MoAtg8 (*Magnaporthe oryzae*, XP_003717877.1), CcAtg8 (*Coprinopsis cinerea*, XP_001833603.1), and ScAtg8 (*Saccharomyces cerevisiae*, NP_009475.1)

To identify the Atg8 homologous protein in *U. virens*, the amino acid sequence of Atg8 (GenBank KZV12988) from *Saccharomyces cerevisiae* was used as a query for the BLASTP search on NCBI. The KDB12146.1 (termed as UvAtg8**)** was hit as the ortholog of *S. cerevisiae* Atg8 with 78% identity and 90% similarity (Fig. 1a). UvAtg8 consists of 121 amino acids. Sequence analysis using motif scan (https://myhits.isb-sib.ch/cgi-bin/motif_scan) revealed that UvAtg8 contained a MAP 1_LC3 domain between 13 and 116 amino acids. The phylogenetic analysis results of the amino acid sequences of Atg8 from *U. virens, F. graminearum*, *Aspergillus niger, Neurospora crassa, Metarhizium brunneum*, *Sclerotinia sclerotiorum, M. oryzae*, *Coprinopsis cinerea*, and *S. cerevisiae,* revealed that UvAtg8 protein was most similar to Atg8 of *M. brunneum* and *S. sclerotiorum*. These results indicated that Atg8 is highly conserved in different species, and also suggested that UvAtg8 may have important functions in *U. virens* (Fig. 1b).

### Disruption and Complementation of *UvATG8*

**Fig. 2:**
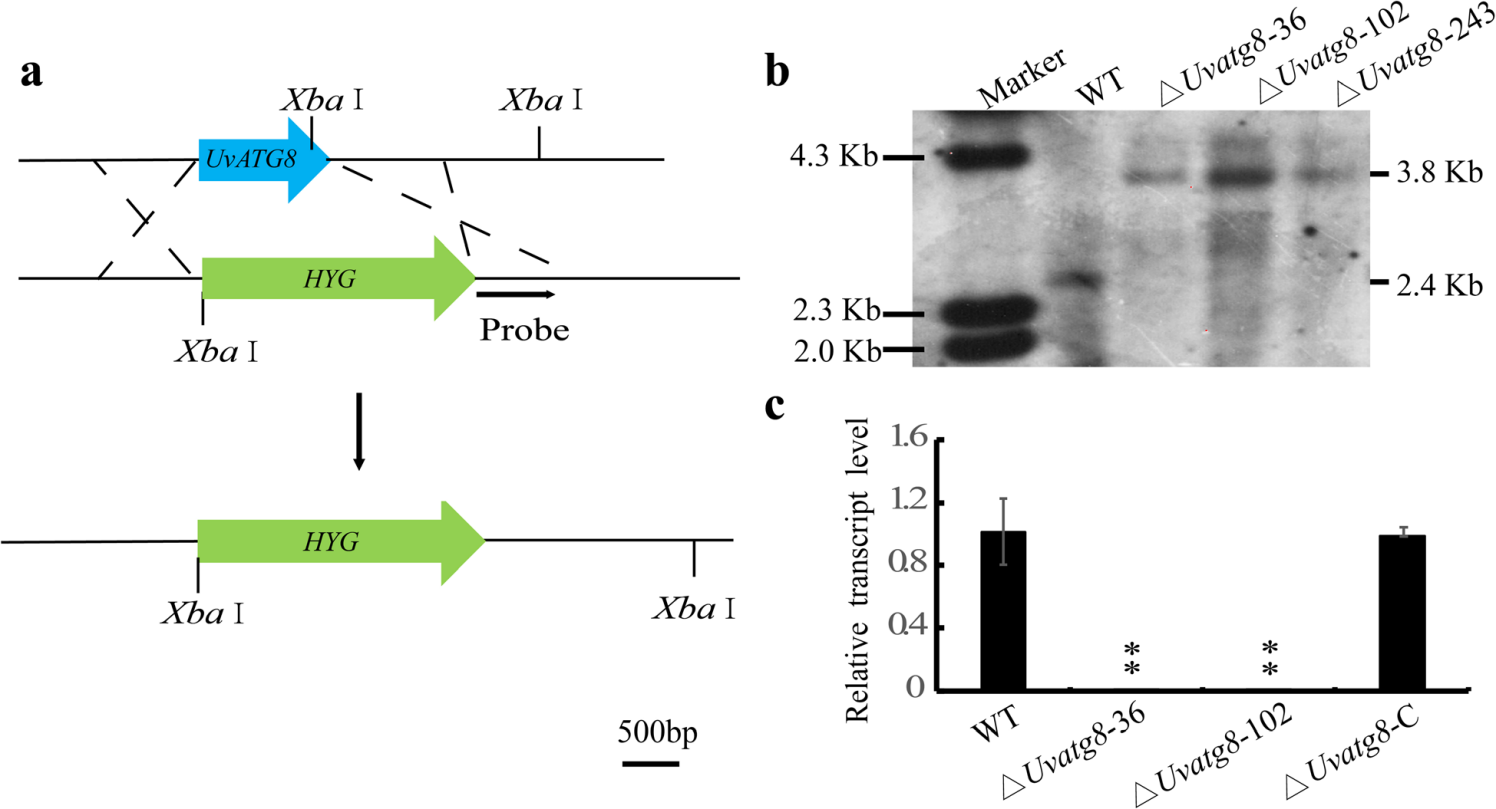
Targeted knockout of *UvATG8* and complement assay of the ∆*Uvatg8* in *Ustilaginoidea virens*. a, Disruption strategy for the *U. virens UvATG8* gene. The *UvATG8* coding region was replaced with the *hygromycin phosphotransferase* gene cassette (*HYG*) by homologous recombination. b, Southern blot analysis of the WT (wild type) and ∆*Uvatg8* mutant strains. The *Xba* I digested genomic DNA from the WT and ∆*Uvatg8* strains were processed for the Southern blotting with the 1 Kb downstream of *UvATG*8 as the probe. c, qRT-PCR analysis of the expression of *UvATG8* in the WT, ∆*Uvatg8*, and ∆*Uvatg8*-C strains. *β-tubulin* gene was used as the endogenous reference gene. The data represent the mean ± SD from three independent replicates. The data were subject to Duncan’s Test and the significant differences were indicated in the figure with two asterisks (**, *p* < 0.005)

To understand the biological functions of *UvATG8*, *UvATG8* deletion mutants were generated by targeted gene replacement in the wild type (WT) HWD-2 strain (Fig. 2a). Southern blot assay was performed to confirm the targeted gene replacement events and excluding ectopic integrations. The appearance of the 3.8 Kb (kilobases) band in the ∆*Uvatg8* mutant, with concomitant loss of the WT 2.4 Kb in *UvATG8* locus, indicated that the gene replacement event was correct (Fig. 2b). The resultant three ∆*Uvatg8* mutants showed comparable phenotypes and formed smaller colonies than the WT, and ∆*Uvatg8*–36 and 102 were chosen for further experiments.

To confirm that the phenotypic differences observed in the ∆*Uvatg8* mutants were all associated with the gene replacement event, a vector pFGL820-*UvATG8* containing a full-length gene copy of *UvATG8* with its native promoter was transformed into ∆*Uvatg8*–36. The resultant ∆*Uvatg8*-C strain was further confirmed using qRT-PCR (quantitative Real-time PCR) assay. The expression levels of *UvATG8* in the WT and ∆*Uvatg8*-C strain were comparable, which indicated that ∆*Uvatg8*-C had rescued the expression of *UvATG8* (Fig. 2c). Moreover, the ∆*Uvatg8*-C strain was similar to the WT strain in colony morphology, suggesting that ∆*Uvatg8*-C functionally complemented the phenotype of ∆*Uvatg8*.

### UvAtg8 Could Sever as a Marker for Autophagy in *U. virens*

**Fig. 3:**
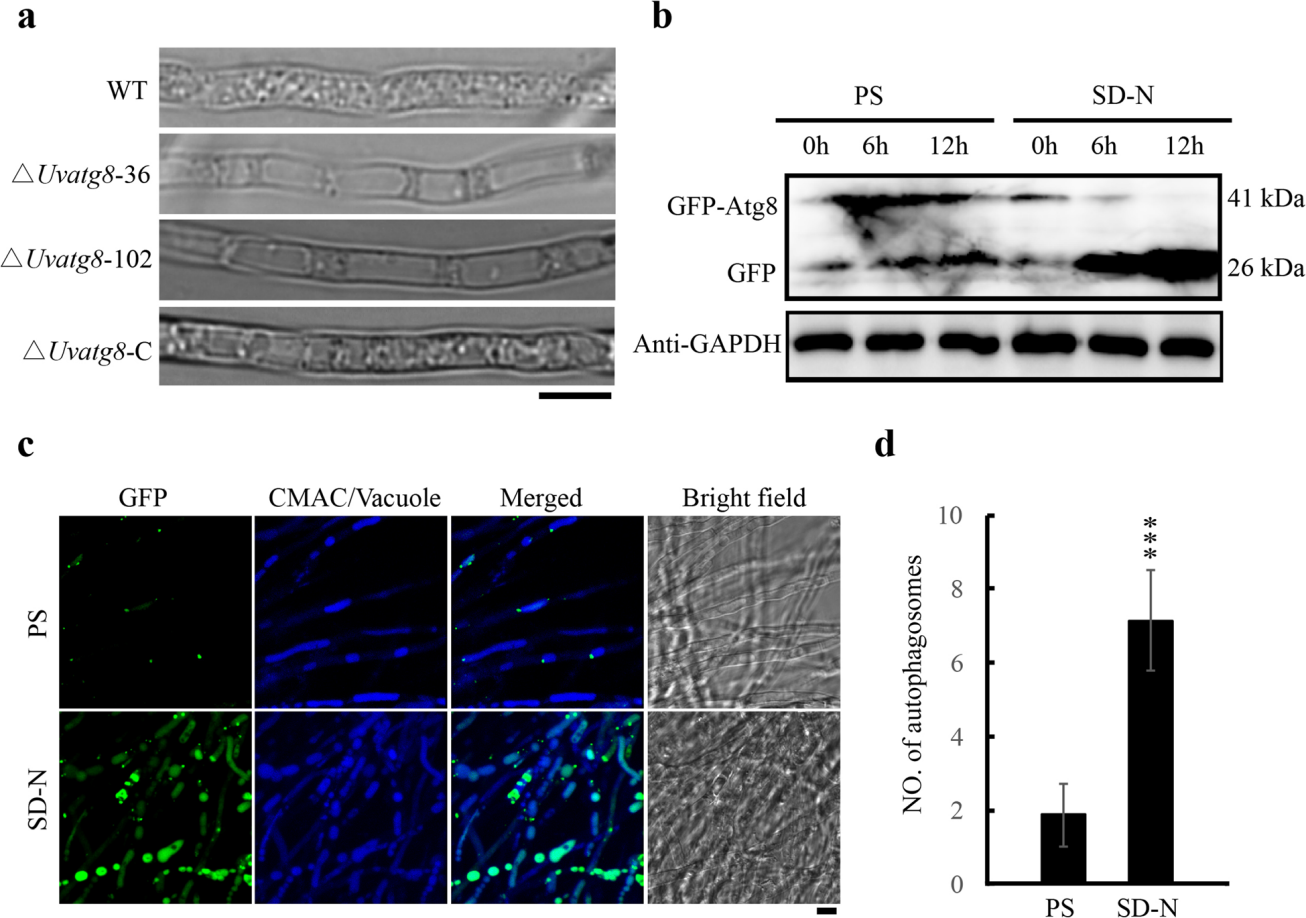
UvAtg8 is essential for the autophagy in *Ustilaginoidea virens*. a, Autophagy was blocked in ∆*Uvatg8* mutants. The strains were cultured in the PS (potato sucrose medium) medium for 3 d, following subjected to SD-N (nitrogen starvation condition) medium for 4 h to determine the autophagic affuxes in the WT and ∆*Uvatg8* strain. The images were taken by bright field microscopy. b, The degradation of the GFP-UvAtg8 was observed under nitrogen starvation condition via Western blot assay with an anti-GFP. GAPDH was used as an internal reference. c, Confocal microscopy image of the *GFP-UvAtg8* strain under nitrogen rich and starvation condition. The indicated strain was cultured in the PS medium for 3 d, and then shifted to an SD-N medium for 12 h. The vacuoles were stained with CAMC (7-amino-4-chloromethylcoumarin) before the observation using confocal laser scanning microscope. Scale bar = 5 μm. d, The number of autophagosomes was counted. The data were subjected to Duncan’s Test and the significant difference was indicated by asterisks (***, *p* < 0.001)

Atg8 is known to be one of the key components of autophagy (Liu et al. 2016; Zong et al. 2016; Hofius et al. 2017). To determine whether *UvATG8* is required for autophagy in *U. virens*, the changes in the process of autophagy in the WT and ∆*Uvatg8*-C strains were determined using microscopy (Fig. 3a). When cultured in SD-N (synthetic dropout medium without nitrogen) liquid medium in the presence of 3 mM PMSF (Phenylmethanesulfonyl fluoride) for 4 h, less than 10% of the vacuoles had a very few autophagic bodies in the ∆*Uvatg8* mutant. In contrast, nearly 65% of the vacuoles in the WT strain exhibited multiple autophagic bodies. These results suggested that the autophagic pathway was blocked in the ∆*Uvatg8* mutant.

To observe the localization of UvAtg8, the *U. virens* strain expressing GFP-UvAtg8 fusion protein under native regulation and as the sole copy of *UvATG8* was generated. The size of GFP-UvAtg8 protein was correct and the morphologies of the *GFP-UvATG8* strain were comparable to the WT strain, indicating that the GFP-UvAtg8 fusion protein was functional in *U. virens*. In order to induce autophagy, the *GFP-UvATG8* strain was cultured in a liquid PS (Potato sucrose) medium for 2 d, and then inoculated into the SD-N medium in the presence of 3 mM PMSF. During the growth process in the PS medium, the GFP signals were weak and in punctate structures in the *GFP-UvATG8* strain. However, following the growth in SD-N medium for 12 h, the GFP fluorescent signals increased and could be easily observed in the CMAC (7-amino-4-chloromethylcoumarin) stained vacuoles (Fig. 3c, d). These results indicated that the GFP-UvAtg8 was localized in the cytoplasm as pre-autophagosomal structures under general conditions, but were predominantly accumulated in the lumen of the vacuoles under nitrogen starvation condition.

Next, the Western blot assay was performed to analyze the expression of GFP-UvAtg8 with anti-GFP antibody under both normal and nitrogen starvation conditions. Based on the predicted sizes and relative mobility, the two detected bands were judged to be the full-length GFP-UvAtg8 and likely the free GFP (Fig. 3b). The stronger GFP band indicated enhanced expression and degradation of the GFP-UvAtg8 in the SD-N medium, and indirectly showed enhanced autophagic response in *U. virens* under nitrogen starvation condition.

Taken together, these results suggested that UvAtg8 is essential for autophagy in *U. virens*, and GFP-UvAtg8 could serve as an appropriate marker for monitoring autophagy in *U. virens* as GFP/RFP-Atg8 in other organisms (Deng et al. 2009; Zong et al. 2016).

### UvAtg8 Plays Important Roles in Vegetative Growth

**Fig. 4:**
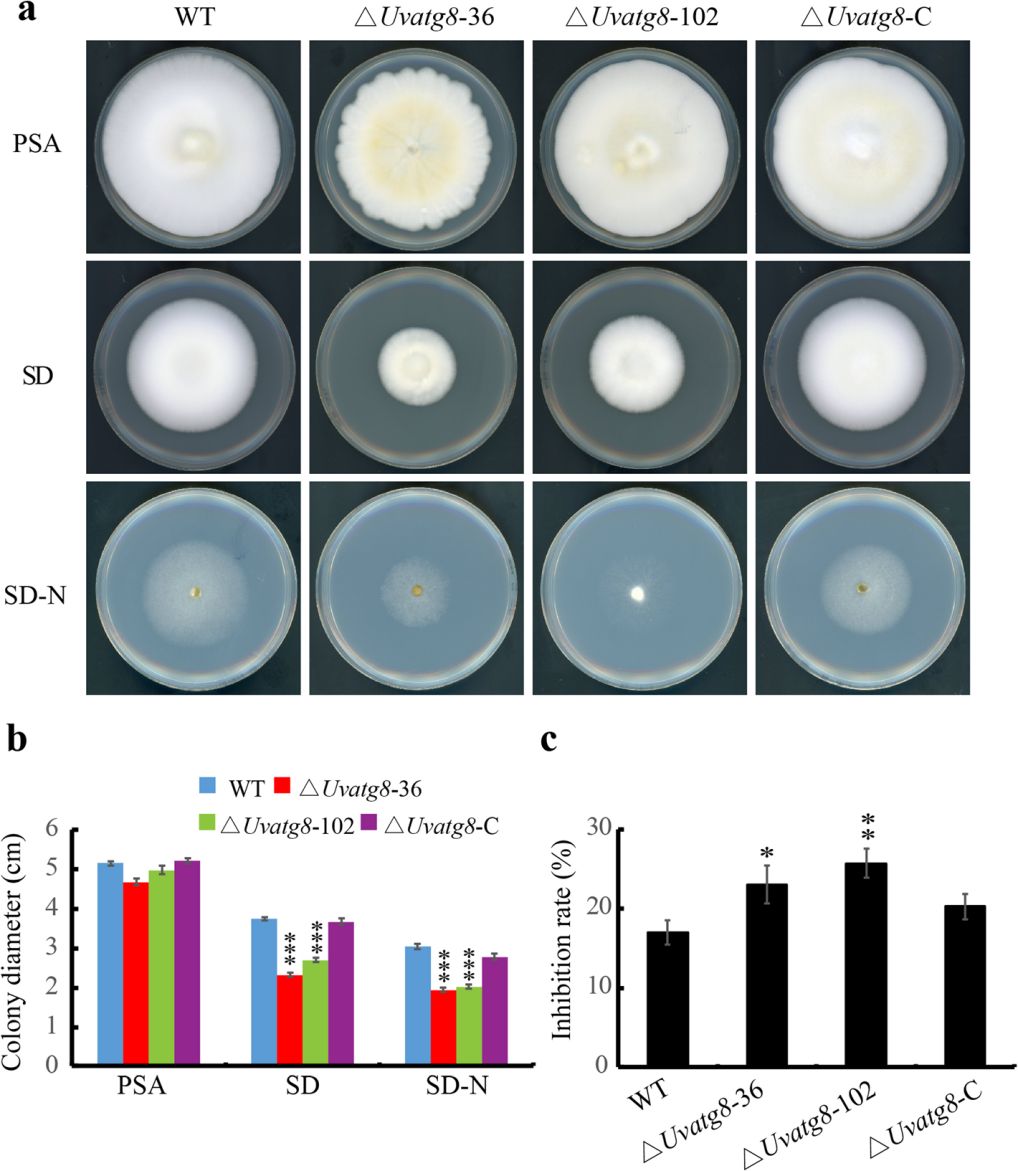
UvAtg8 plays important roles in the fungal growth. a, Radial growth of ∆*Uvatg8* was reduced. Colonies of the WT, ∆*Uvatg8*, and ∆*Uvatg8*-C strains were grown on the PSA、SD and SD-N medium in dark for 15 d and then imaged. b, Statistical analysis of colonies diameters of test strains on the PSA, SD and SD-N plates. c, Statistical analysis of inhibition rate of test strains under nitrogen starvation condition by comparing the fungal growth on the SD-N and SD medium. Three independent biological experiments were performed with three replicates each time. The data were subjected to Duncan’s Test and the significant differences were shown in the figure with asterisks (*, *p* < 0.01; **, p < 0.005; ***, p < 0.001)

Because the ∆*Uvatg8* mutants had formed smaller colonies than the WT strain, the vegetative growth of the WT, ∆*Uvatg8*, and ∆*Uvatg8*-C strains were determined using different mediums. The ∆*Uvatg8* strain was slightly reduced in mycelia growth when compared with that of the WT strain on the PSA. However, the mycelia growths of the ∆*Uvatg8* strain on the SD and SD-N medium were significantly reduced (Fig. 4a,b). Notably, the inhibition rate of the ∆*Uvatg8* strain was significantly increased when compared with that of the WT strain under the nitrogen starvation condition, which indicated that its defects in hypha growth were partially dependent on the nutrient conditions (Fig. 4a,c). In contrast, the mycelia growth and colony morphology were rescued in the ∆*Uvatg8*-C strain (Fig. 4), suggesting that UvAtg8 plays important roles in the vegetative growth of *U. virens.*

### UvAtg8 Is Involved in Various Stress Responses

**Fig. 5:**
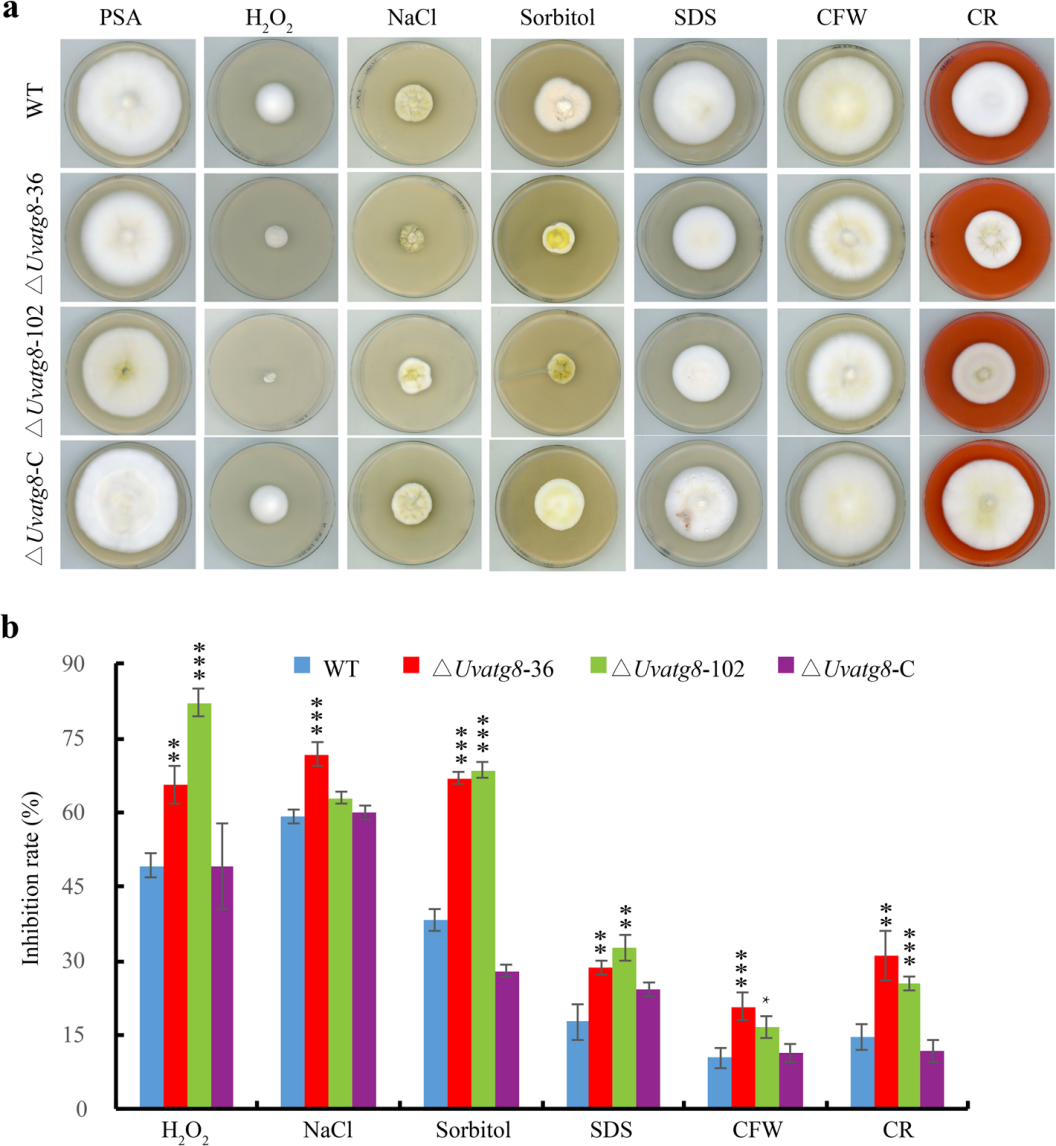
Defects of the ∆*Uvatg8* mutants in response to different stresses. a, The represent strains were cultured on regular PSA plates or PSA plates with 0.03% H_2_0_2_, 0.4 M NaCl, 0.7 M sorbitol, 0.03% Sodium dodecyl sulfate (SDS), 200 μg/mL Calcofluor white (CFW), 240 μg/mL Congo red (CR) plates. Typical cultures were photographed after incubation for 15 d at 28 °C. b, Statistical analysis of inhibition rate of test strains under different stress conditions. The diameters of colonies were detected and analyzed. Similar results were obtained by three repeated experiments. The error bars represent the standard deviation and the asterisk represents the significant difference compared to the WT strain under the same conditions (*, p < 0.01; **, p < 0.005; ***, p < 0.001)

Autophagy is known to be related to several stress responses (Yin et al. 2019). However, it remains unclear whether *UvATG8* regulates stress responses of *U. virens.* Therefore, the sizes of ∆*Uvatg8* colonies were measured under different stress conditions, including oxidative, hyperosmotic, and cell wall stresses. In the presence of 0.03% H_2_O_2_, the growth was reduced by 50% in the WT strain, but approximately 75% in the ∆*Uvatg8* mutant after incubation for 15 d (Fig. 5). These results indicated that the ∆*Uvatg8* was sensitive to oxidative stress. Similarly, both the ∆*Uvatg8*–36 and 102 had shown significantly increased inhibitory effects when compared with the WT strain on the medium with 0.4 M NaCl or 0.7 M Sorbitol (Fig. 5), thereby suggesting that *UvATG8* contributed to the adaptions to the hyperosmotic stresses. In addition, the deletion of *UvATG8* resulted in decreased tolerance to different cell wall stresses (Fig. 5), including 0.03% Sodium dodecyl sulfate (SDS), 200 μg/mL Calcofluor white (CFW), and 240 μg/mL Congo Red (CR), in *U. virens*. The complementation strain ∆*Uvatg8*-C showed similar phenotypes as the WT strain under all of the observed stress conditions. All the aforementioned results indicated that UvAtg8 is involved in the adaptions to oxidative, hyperosmotic, and cell wall stresses in *U. virens*.

### UvAtg8 Is Required for Pathogenesis in *U. virens*

**Fig. 6:**
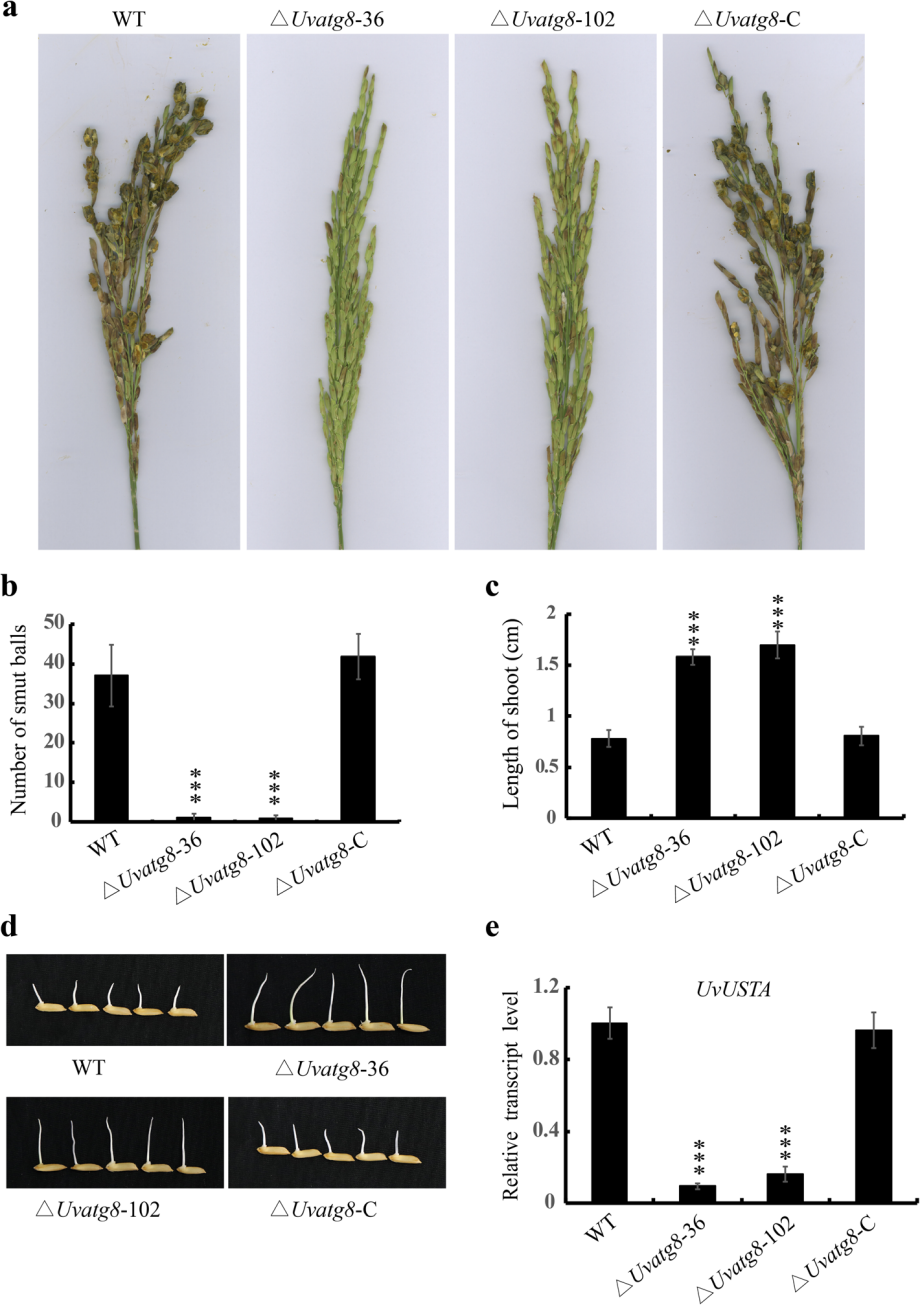
Virulence assays of the ∆*Uvatg8* mutants. a, Disease symptoms caused by the WT, ∆*Uvatg8*, and ∆*Uvatg8*-C strains on 21 d after inoculation. b, Statistical analysis results of the average number of false smut balls per spikelet. At least three independent biological experiments were performed with 30 inoculated panicles each time. c, Culture filtrates of the ∆*Uvatg8* mutants displayed less inhibitory of rice seedling germination. The shoot lengths were measured and statistically analyzed after 7 d in 28 °C illumination incubators. d, The growths of the rice shoots of the indicated strains were treated with testing culture filtrates. More than 100 rice seeds were sterilized with 0.1% potassium permanganate, then shifted to 35 mL culture filtrates at 28 °C for 7 d. e, qRT-PCR analysis of the expression of *UvUSTA* in the WT, ∆*Uvatg8*, and ∆*Uvatg8*-C strains. *β-tubulin* gene was used as the endogenous reference gene. Three independent experiments (*n* = 100) were carried out (mean ± SD). *** indicates p < 0.001

To investigate the roles of *UvATG8* in the virulence of *U. virens*, infection assays were performed. The WT, ∆*Uvatg8*–36 and 102, and ∆*Uvatg8*-C strains were inoculated into panicles of the susceptible rice cultivar Wanxian 98. The formation of rice false smut balls were calculated 3 weeks after the inoculations. Remarkably, the numbers of smut balls in the ∆*Uvatg8*–36 and 102 strains were significantly lower than those in the WT and complementation strains (Fig. 6a). Therefore, deletion of *UvATG8* significantly attenuated *U. virens* virulence, suggesting that *UvATG8* is a key regulator of the pathogenicity of *U. virens.*

*U. virens* not only occupies rice grains, but also produces compounds, eg. Ustilaginoidins O, E, F and isochaetochromin B2, which are toxic to rice seeds (Lu et al. 2015). In order to determine the inhibitory effect of toxic compounds on the germination of rice seeds, the filtrates were isolated from the PS cultures of 7 d old WT, ∆*Uvatg8*–36 and 102, and ∆*Uvatg8*-C strains to treat rice seeds. As shown in Fig. 6d, the shoots of the rice treated by the filtrate of the ∆*Uvatg8* strain culture was significantly longer than those of the WT and ∆*Uvatg8*-C strains. Furthermore, the expression level of *UvUSTA*, which is a member of the gene cluster responsible for ustiloxin synthesis, in the deletion mutant of *UvATG8* was lower than that of the WT strain (Fig. 6e) (Tsukui et al. 2015; Zheng et al. 2016). These findings suggested that *UvATG8* may have produced fewer toxic compounds which inhibit the shoot growth of rice shoots during rice seed germination.

### Serious Virulence Defects in *∆UvAtg8* Were Mainly Caused by the Highly Decreased Formation of Secondary Spores

**Fig. 7:**
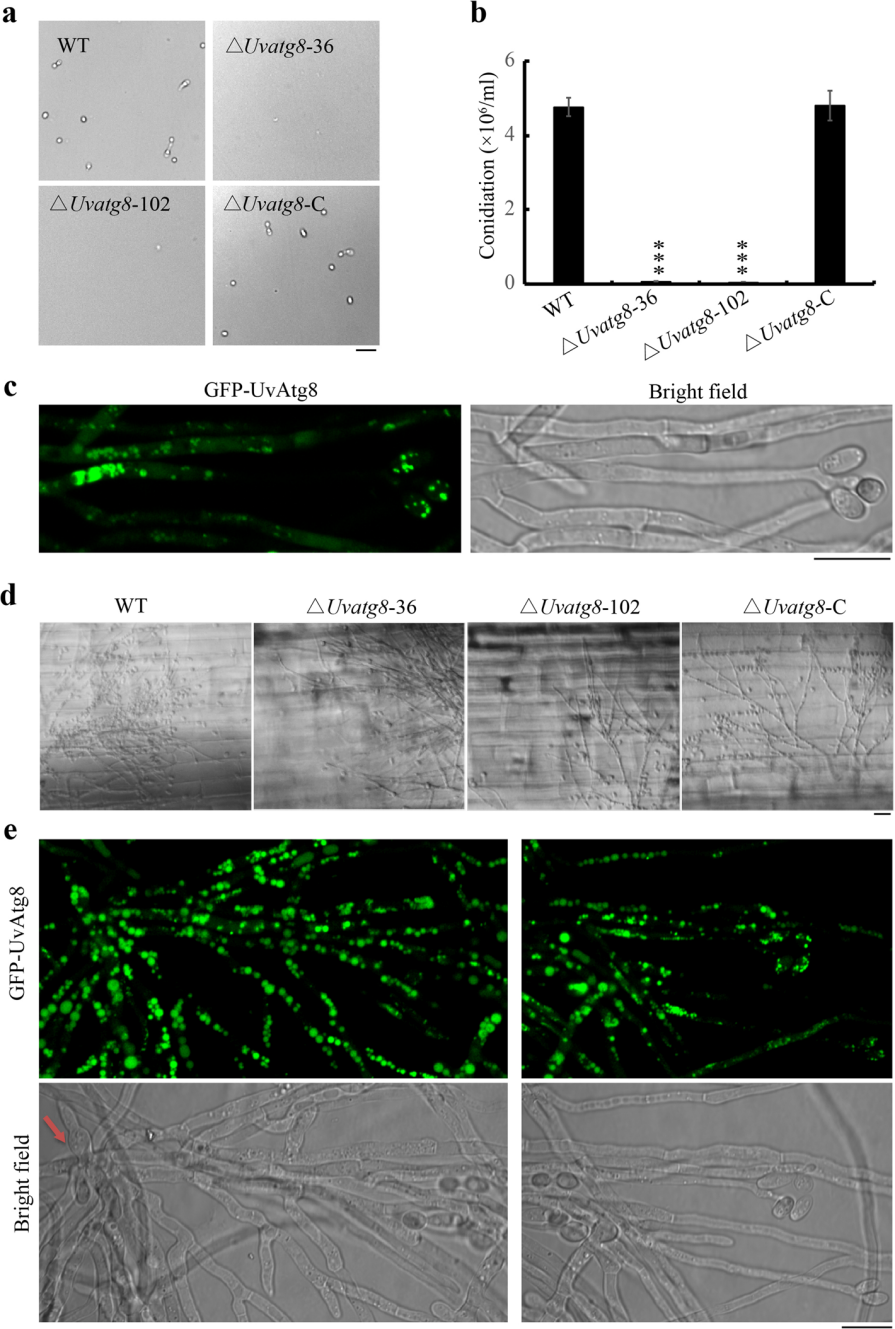
Deletion of *UvATG8* resulted in decreased conidiation. a and b, Conidiation of the tested strains. The strains were cultured in 50 mL of PS medium with 170 rpm at 28 °C for 7 d prior to the observation. Three repetitions were performed, with similar results obtained. c, The expression of GFP-UvAtg8 during conidiation. d, Conidial germination of *U. virens* on rice sheath. 5 μL conidial droplets (1 × 10^6^ conidia/mL) were inoculated and incubated at 28 °C for 3 d. Photographs were taken under an Olympus BX53 microscope equipped with bright field optics. e, The GFP-UvAtg8 was highly expressed, and autophagy had occurred during the secondary spore formation stage. The images were obtained using a confocal laser scanning microscope. Red arrow showed the conidial germination. Scale bar = 2.5 μm

To further investigate the serious virulence defects caused by deletion of *UvATG8,* the infection processes of the WT, ∆*Uvatg8*, and ∆*Uvatg8*-C strains were determined. Although the structures of the conidia appeared to be normal, the conidial production of ∆*Uvatg8* mutant was highly reduced in comparison with that of the WT and ∆*Uvatg8*-C strains (Fig. 7a,b). Whereas the WT strain had formed 4.8 × 10^6^ conidia/mL in 7 d old cultures, the ∆*Uvatg8* mutants had produced less than 0.1 × 10^6^ conidia/mL under the same conditions. This may have been the reason for the virulence deficiency of the ∆*Uvatg8* strain. Furthermore, the GFP-UvAtg8 was likely presented in the vacuoles during conidiation (Fig. 7c), indicating that autophagy had occurred at that stage. However, when the same concentrations of conidia were inoculated, the infection efficiency of the ∆*Uvatg8* strain was still observed to be highly decreased (Fig. 6a,b). Therefore, the results showed that, with the exception of the slightly slower growth and lower conidiation in the ∆*Uvatg8* strain, there were definitely other reasons for the virulence deficiency of the ∆*Uvatg8* strain*.*

Next, the germination of the conidia from ∆*Uvatg8* mutant was detected. There were no significant differences observed in the germination of conidia between the WT strain and ∆*Uvatg8* mutant. However, it should be noted that the formation of secondary spores was highly reduced in the ∆*Uvatg8* mutant on the rice surface (Fig. 7d). During pathogenic processes of *U. virens,* the formation of secondary spores tends to greatly increase the amount of inoculation available to infect rice plants (Fan et al., 2013). Therefore, the highly reduced formation of secondary spores may have been the main reasons for the pathogenicity defects in the ∆*Uvatg8* mutant. Meanwhile, the expression of GFP-UvAtg8 was highly induced and targeted to the vacuoles (Fig. 7e, S1), suggesting that the UvAtg8 mediated autophagy occurrences and played an important role in formation of secondary spores during the infection of rice.

## Discussion

*U. virens* has become one of the most devastating rice pathogens worldwide. Autophagy plays an important role in the maintenance of normal cell differentiation and development. However, at present there is no information available regarding the function of autophagy in *U. virens* virulence. In this study, through gene deletion, complementation, and localization analyses, it was successfully confirmed that UvAtg8 is required for autophagy*,* and it was indicated that GFP-UvAtg8 could be a useful marker for the study of autophagy in *U. virens.* Moreover, *UvATG8* was also identified as key regulator of the pathogenicity of *U. virens* via its function in growth, various stresses responses, conidiation, and the formation of secondary spores during pathogenesis.

Atg8 is a key component of the autophagy pathway. During autophagy, Atg8 is localized to the autophagosomes, and then internalized in the vacuoles or lysosomes after fusion between the autophagosomes and vacuoles/lysosomes (Liu et al. 2016; Zong et al. 2016; Hofius et al. 2017). In this study, we found that GFP-UvAtg8 could be a marker for autophagy in *U. virens.* First, *UvATG8* is essential for autophagy*,* and the deletion of *UvATG8* resulted in blocked autophagic pathway in *U. virens*. These results suggested that the functions of UvAtg8 are conserved among various species. Therefore, fluorescent-marker-tagged UvAtg8 may be used to visualize the occurrences of autophagy similar to Atg8 in other organisms (Zong et al. 2016; Deng et al. 2009). Second, the *GFP-UvATG8* (with only one copy of *UvATG8*) functions properly in *U. virens,* and the fluorescence intensities and localization of GFP-UvAtg8 were changed by the autophagy-induced conditions. The vegetative growths of the *GFP-UvATG8* strain are comparable to the WT strain under both normal and nutrient starvation conditions. Moreover, the expression of GFP-UvAtg8 was highly induced in the SD-N medium (nitrogen starvation medium), and was considered to have induced autophagy. Furthermore, Western blot assay could be performed to show the level of GFP-UvAtg8 to monitor the quantity of the autophagy in *U. virens.* In conclusion, GFP-UvAtg8 could serve as a good marker for monitoring the autophagy in *U. virens.*

Although the genomic sequences of *U. virens* have been released, there are only a small number of pathogenicity-related genes which have been functionally characterized and experimentally verified as pathogenic factors (Qiu et al. 2019). These pathogenic factors include low-affinity iron transporter Uvt3277, hypothetical protein UvPro1, adenylate cyclase UvAc1, phosphodiesterase UvPdeH, effector Scre2 (Uv-1261), Bax inhibitor UvBI-1, two protein kinases UvPmk1 and UvCDC2, and transcription factor UvCom1 (Zheng et al. 2017; Xie et al. 2019; Tang et al. 2019; Lv et al. 2016; Guo et al. 2019; Fang et al. 2019; Fan et al. 2019; Chen et al. 2019). In this study, *UvATG8* was characterized as a pathogenic factor. Loss of *UvATG8* resulted in impaired pathogenesis. The slightly hypha growth defects and lower conidiation may contribute to the pathogenicity defects in *U. virens.* However, these may not be the major causes of the serious defects in virulence in the *∆UvAtg8* mutant, since the infection efficiency of the ∆*Uvatg8* strain was still highly decreased in the inoculation assays with the same concentrations of conidia. *U. virens* has been reported to have epiphytic characteristics (Fan et al., 2013). In the epiphytic colonization of *U. virens,* the rapid production of large numbers of secondary spores greatly increases the amount of inoculation potential of *U. virens* on rice spikelets, which will subsequently lead to the increased occurrences of rice smut disease (Fan et al., 2013). In the *∆UvAtg8* mutant, the generation of secondary spores was highly reduced. Moreover, the expression of the GFP-UvAtg8 was highly induced during secondary spore formation, indicating that the UvAtg8-mediated autophagy regulates secondary spore formation to promote the infection of *U. virens*. In addition to the spore formation, UvAtg8 can be also important for the infection processes, because *U. virens* may depend on autophagic utilization of its own nutritional storage to initiate infection before getting access to host nutrients. Taken together, UvAtg8 affects the pathogenicity by regulating the hypha growth, conidiation, and, most importantly, the formation of secondary spores in *U. virens*.

In conclusion, our study indicated that UvAtg8 is necessary for the fungal growth, stress responses, conidiation, secondary spore formation, and pathogenicity in *U. virens*. In addition, GFP-UvAtg8 provided a good marker for monitoring of the autophagy in *U. virens*. Furthermore, the results of this study facilitated a deeper understanding of the pathogenic molecular mechanism of *U. virens*, that autophagy plays an important role in the pathogenicity of *U. virens.*

## Materials and Methods

### Sequence Analysis

The sequences of the genes and proteins used in this study were downloaded from the National Center for Biotechnology Information (NCBI, https://www.ncbi.nlm.nih.gov/). The protein sequence alignments were processed using EPSript 3.0, and the phylogenetic analyses were performed using MEGA 5.0 with a neighbor-joining algorithm (Robert and Gouet 2014).

### Fungal Strains and Culture Media

The *U. virens* WT strain HWD-2 was kindly provided by Professor Junbin Huang from Huazhong Agriculture University (Hubei, People’s Republic of China). The routine culturing of the *U. virens* strain was carried out on potato sucrose agar (PSA, potato 200 g/L, sucrose 20 g/L, and agar 20 g/L) medium at 28 °C. The DNA and RNA samples were collected from mycelia which had been cultured in the PS (potato 200 g/L, sucrose 20 g/L) medium for 7 d.

For the conidia concentrations, six agar plugs with mycelium were inoculated into a 100 mL flask, then 50 mL of liquid PS medium was added. The agar plugs were vibrated at 170 rpm for 7 d at 28 °C. The filtration of the samples was used to measure the conidia concentration and conidia germination. The conidia were germinated on rice sheath.

To test the sensitivity to various abiotic stress conditions, the *U. virens* strains were measured after grown on the PSA medium and PSA medium with 0.03% H_2_O_2_, 0.4 M NaCl, 0.7 M Sorbitol, 0.03% SDS, 200 μg/mL CFW or 240 μg/mL CR at 28 °C for 15 d. For the nutrient starvation, the SD (Yeast nitrogen base without amino acids and 1.7 g/L, Ammonium sulfate, 5 g/L glucose 20 g/L) medium and SD-N (Yeast nitrogen base without amino acids and 1.7 g/L, glucose 20 g/L) medium was used. The formula of the inhibition rate was as follows: Inhibition rate = (average of strain colony diameters on the PSA – the average of strain colony diameters on the PSA with different chemicals)/average of the strain colony diameters on the PSA × 100%. All of the experiments were repeated three times with three replicates each time.

### Construction of the Δ*Uvatg8* Strains and Complementation Analyses

The *UvATG8* gene (*UV8b_6970*) deletion mutant was generated using a one-step gene replacement strategy. Briefly, approximately 1 Kb upstream and downstream flanking sequences of *UvATG8* were PCR amplified and ligated sequentially to flank of the *hygromycin phosphotransferase* gene cassette in pFGL821 (Addgene, 58,224). Primers used to amplify the flanking sequences of the *UvATG8* gene were shown in Table S1. The final plasmid construct was confirmed by sequencing, and subsequently introduced into the HWD-2 strain by *Agrobacterium tumefaciens-*mediated transformation (ATMT) to replace the *UvATG8* gene (Tang et al. 2019). For complementation analysis, the full-length genomic copy with the promoter of *UvATG8* was amplified with UvATG8-F and UvATG8-R (Table S1), and then inserted into pFGL822 (Addgene, 58,226). The resultant plasmid was transformed into the Δ*Uvatg8* strain.

The correct transformants of the Δ*Uvatg8* and complementation assay in this study were ascertained using Southern blot and/or qRT-PCR analysis. For the Southern blot analysis, the genomic DNA samples from the WT and ∆*Uvatg8* strains were extracted and digested with *Xba* I. The digested products were then separated through 0.8% agarose gel and mounted onto positively charged nylon membrane (GE Healthcare, Buckinghamshire, UK). Then, the DIG-labeled *UvATG8* probe, which was amplified from the genomic DNA of the WT strain using the primers UvATG8-probeF/R (Table S1), was used to hybridize with the *Xba* I digested genomic DNA of the WT and ∆*Uvatg8* mutants. The hybridization process was performed following manufacturing instructions provided by Amersham™ AlkPhos Direct Labeling Reagents (GE Healthcare, Buckinghamshire, UK).

### qRT-PCR Analysis

In regard to the qRT-PCR, the total RNA from the mycelium cultured in PS medium was extracted using the Fungal RNA Kit 200 (OMEGA). The reserve transcriptional analysis was performed using Primescript™ RT reagent kit with gDNA Eraser and TB Green™ Premix Ex Taq™ (Tli RnaseH Plus) (Takara) as the instrument. The *β-tubulin* gene was used as the endogenous reference gene (Table S1), and the relative expression level of the gene was calculated using the 2-^∆∆^Ct method. Three biological replicates were performed to calculate the mean and standard deviation.

### Generation of the *GFP-UvATG8* Strain and Western Blot Analysis

For generating the plasmid vector to express the *GFP-UvATG8*, approximately 1 Kb of downstream flanking sequences of *UvATG8* were PCR amplified and ligated to *pFGL821*. Then, 1 Kb of promoter region of *UvATG8*, *eGFP*, and the CDS of *UvATG8* were inserted into resulted construct to obtain the *GFP-UvATG8* (Table S1). The sequences of the plasmid were confirmed by sequencing and were subsequently introduced into the WT strain by ATMT to replace *UvATG8*. The transformants were confirmed by PCR and epifluorescence microscopic observation.

For the induction of autophagy, the *GFP-UvATG8* strain was grown in the PS (PS, potato 200 g/L, sucrose 20 g/L) medium for 3 d, followed by starvation in SD-N (Yeast nitrogen base without amino acids and 1.7 g/L, glucose 20 g/L) medium for 0, 6, and 12 h, respectively. The mycelia grown in the PS medium were used as control. Total proteins were extracted from the mycelia grown under the requisite conditions as described (Liang et al. 2018). The protein samples were quantified using a BCA protein content assay kit (Cat. No. C503021, Beyotime, China). Equal total proteins were loaded and separated on a 12% SDS-PAGE (Sodium dodecylsulphate polyacrylamide gel electrophoresis) (Bio-Rad, Hercules, USA), which were then transferred to a PVDF (Polyvinylidene fluoride) membrane. The anti-GFP antibody (Cat. ab32146, Abcam, UK) and an anti-rabbit IgG (Cat. ab205718, abcam, UK) were used for the blotting processes. The samples were also hybridized with the monoclonal anti-GAPDH antibody (Cat. No. R1208–3, HuaBio, China) to be an internal reference. All of the experiments were repeated three times.

### Live Cell Imaging and Image Processing

To visualize the vacuole, the mycelia were incubated with 10 μM CellTracker™ Blue CMAC Dye (Molecular Probes C2110) for 20 mins at 28 °C. The samples were washed with water prior to the microscopic observation.

The live cell imaging of this study was performed using a Zeiss LSM 700 inverted confocal microscope (Carl Zeiss, Inc) equipped with a Plan-Apochromat 63 (NA 1.40) oil immersion lens. EGFP and CMAC excitations were performed at 488 nm (Em. 505–530 nm) and 405 nm (Em. 430–470 nm), respectively. The images were processed in the Image J program from National Institutes of Health (http://rsb.info.nih.gov/) and arranged in Adobe Illustrator CS6.

### Plant Infection and Toxicity Assays

For the infection assay, a mixture of mycelia and conidia (10^6^ conidia/mL) was broken down for 3 min in a juice blender. Then, the conidia suspension was adjusted to 1× 10^6^ conidia/mL to inject the panicles of selected rice plants (*Oryza sativa* L., cultivar Wanxian98) at the booting stage (6–7 d before heading). The inoculated plants were grown in a growth chamber under 12 h light/dark conditions, at a temperature of 25 °C and with 90% humidity. Smut balls were assessed and recorded by scanning the leaves at 21 d post inoculation. The values were represented as the mean ± SD from three independent experiments, with more than 30 inoculated panicles each time. Toxicity assays with culture filtrates were performed as described using Wanxian98 seeds (Zheng et al. 2016). All of the experiments in this section were performed in three independent biological experiments with three replicates in each test.

## Supplementary information

**Additional file 1: Table S1.** Primers used in this study.

**Additional file 2: Fig. S1.** The GFP-UvAtg8 was highly expressed during the secondary spore formation stage on rice.

## Data Availability

The datasets supporting the conclusions of this article are included within the article and its additional files.

## Abbreviations

ATG: autophagy-related
CMAC: 7-amino-4-chloromethylcoumarin;
CFW: Calcofluor white
CR: Congo red
d: days
Kb: kilobases
*F. graminearum*: *Fusarium graminearum*
GFP: green fluorescent protein
h: hours
*M. oryzae*: *Magnaporthe oryzae*
mins: minutes
PMSF: Phenylmethanesulfonyl fluoride
PVDF: Polyvinylidene fluoride
PS: potato sucrose medium
PSA: potato sucrose agar medium
*S. cerevisiae*: *Saccharomyces cerevisiae*
SD-N: synthetic dropout medium with nitrogen
SDS: Sodium dodecyl sulfate
SDS-PAGE: sodium dodecylsulphate polyacrylamide gel electrophoresis
*Ustilaginoidea virens*: *U. virens*
WT: wild type.
