# Supplementary material for: UvAtg8-Mediated Autophagy Regulates Fungal Growth, Stress Responses, Conidiation, and Pathogenesis in Ustilaginoidea virens
Source: Rice (N Y). 2020 Aug 12;13:56. doi: 10.1186/s12284-020-00418-z (PMC7423828; doi:10.1186/s12284-020-00418-z)
Supplement: Supplementary file 1 — Additional file 1: Table S1. Primers used in this study. [file 12284_2020_418_MOESM1_ESM.doc]

### Table S1. Primers used in this study.

| **Primer** | **Sequence (5’-3’)** | **Application** |
| --- | --- | --- |
| UvATG8-3F | CCGGAACCAGTCGACCTGCAGAATTCGCGCCCTGATCTCTG | Amplifying *UvATG8* 5' flank sequence for gene deletion |
| UvATG8-3R | TTGTAAGCGTTAATCAAGCTTAATCATCCGACAGACAGCCC |
| UvATG8-5F | TATGGAGAAACTCGAGAATTCATCGCTGACTACATATCGCGTTC | Amplifying *UvATG8* 3' flank sequence for gene deletion |
| UvATG8-5R | GACTCTAGAACTAGTGGATCCGACTACCAAAAGACTAGAAGCAGACTTT |
| UvATG8-TR | ACCTCCACCAACCAACCACCA | Transformants screening |
| UvATG8-F | CCGCTCGAGGCATCCAGGTTGACGGTGTTGGC | Δ*Uvatg8* complementation assay |
| UvATG8-R | CGCGGATCCCTCGTGTGTTTCGATCCTACC |
| UvATG8-GFP-5F | TATGGAGAAACTCGAGAATTCATCGCTGACTACATATCGCGTTC | Construction of *GFP:UvATG8* |
| UvATG8-GFP-5R | CTTGCTCACCATGACTACCAAAAGACTAGAAGCAGACTTT |
| GFP-ATG8-F | TGGTAGTCATGGTGAGCAAGGGCGAGG | Construction of *GFP:UvATG8* |
| GFP-ATG8-R | TTGCTTCGCTTGTACAGCTCGTCCATGCC |
| ATG8-F | GAGCTGTACAAGCGAAGCAAATTCAAGGACGAG | Construction of *GFP:UvATG8* |
| ATG8-R | GACTCTAGAACTAGTGGATCCTTAGCCCTCTTGGAAAAAACCA |
| qUvATG8-F | TCAAGGACGAGCATCCCTTC | Amplifying *UvATG8* for RT-PCR  Amplifying *UvUSTA* for RT-PCR |
| qUvATG8-R  qUvUSTA-F  qUvUSTA-R | GTCAAATCTGACGGCACCAG  AGTTTTCGCTCATCTCCATCTTG  CGCTTTGCCATTACATTCTCCT |
| *β-tubulin* F | GGCGTTTACAATGGCACTTC | Analysis the expression level of *β-tubulin* gene of *U. virens* |
| *β-tubulin* R | CGGAACAGTTGACCAAAAGG |
| UvATG8-probeF | TTCGCGCCCTGATCTCTGTC | Amplification of probe for southern blotting and transformants screening |
| UvATG8-probeR | CCACAATAGTCACCGAGAGAGGT |
