# Supplementary material for: UvAtg8-Mediated Autophagy Regulates Fungal Growth, Stress Responses, Conidiation, and Pathogenesis in Ustilaginoidea virens
Source: Rice (N Y). 2020 Aug 12;13:56. doi: 10.1186/s12284-020-00418-z (PMC7423828; doi:10.1186/s12284-020-00418-z)
Supplement: Supplementary file 2 — Additional file 2: Fig. S1. The GFP-UvAtg8 was highly expressed during the secondary spore formation stage on rice. [file 12284_2020_418_MOESM2_ESM.pptx]

## Slide 1
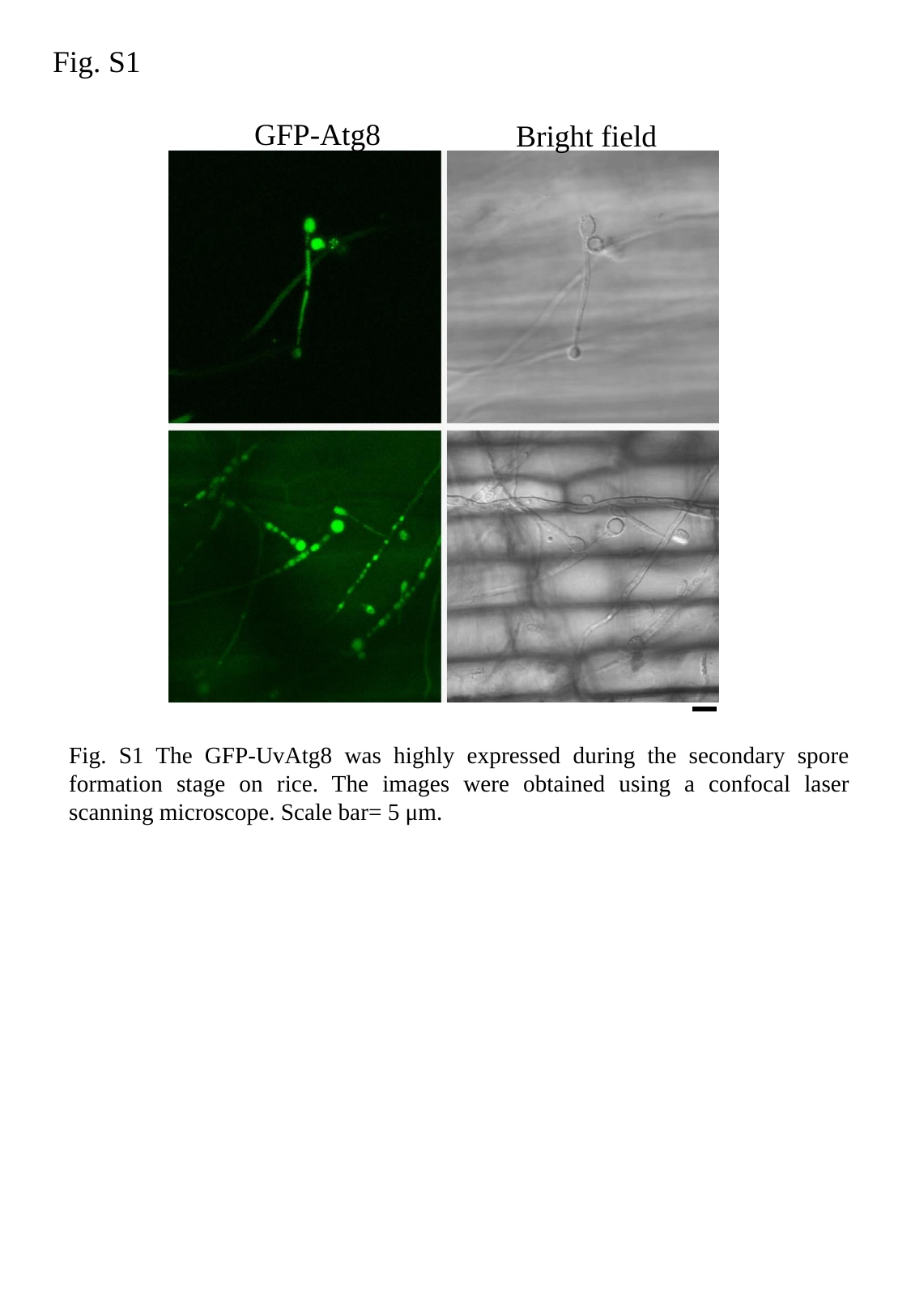

Fig. S1
GFP-Atg8
Bright field
Fig. S1 The GFP-UvAtg8 was highly expressed during the secondary spore formation stage on rice. The images were obtained using a confocal laser scanning microscope. Scale bar= 5 μm.
